# Advancing public health: enabling culture-fair and education-independent automated cognitive assessment in low- and middle-income countries

**DOI:** 10.3389/fpubh.2024.1377482

**Published:** 2024-06-28

**Authors:** Desalegm Garuma, Dheeraj Lamba, Teklu Gemechu Abessa, Bruno Bonnechère

**Affiliations:** ^1^Department of Psychology, College of Education and Behavorial Sciences, Jimma University, Jimma, Ethiopia; ^2^REVAL Rehabilitation Research Center, Faculty of Rehabilitation Sciences, Hasselt University, Diepenbeek, Belgium; ^3^Department of Physiotherapy, Faculty of Medical Sciences, Institute of Health, Jimma University, Jimma, Ethiopia; ^4^Department of Special Needs and Inclusive Education, Jimma University, Jimma, Ethiopia; ^5^Technology-Supported and Data-Driven Rehabilitation, Data Sciences Institute, Hasselt University, Diepenbeek, Belgium; ^6^Centre of Expertise in Care Innovation, Department of PXL–Healthcare, PXL University of Applied Sciences and Arts, Hasselt, Belgium

**Keywords:** cognition, LMIC, assessment, digital health, public health

## 1 Introduction

Cognitive assessment plays a crucial role in understanding cognitive functioning and detecting impairments or changes in cognitive abilities (1). It serves as an invaluable tool in evaluating an individual's cognitive status, aiding in clinical diagnoses, monitoring treatment progress, and informing intervention strategies (2). In low- and middle-income countries (LMICs), where a significant portion of the global population resides, access to reliable cognitive assessment tools is essential for identifying cognitive impairments and promoting cognitive wellbeing (3, 4).

However, the existing cognitive assessment tools used in LMICs are often limited by their heavy reliance on language, education and cultural factors (5). Traditional assessment methods typically involve standardized tests and questionnaires that are influenced by the educational background and cultural context of the individuals being assessed. These tools may inadvertently introduce bias and hinder accurate evaluation, particularly in populations with limited educational opportunities or diverse cultural backgrounds (6). This is particularly concerning given that a low level of education is associated with an increased risk of dementia, especially in LMICs (7).

The reliance on education and culture as prerequisites for cognitive assessment poses significant challenges in LMICs. Limited access to quality education and variations in cultural norms and practices can greatly impact individuals' performance on cognitive tests (8, 9). This dependence exacerbates disparities in assessing cognitive abilities, further worsening health inequalities (10, 11).

Another potential problem arises from the disparities in medical resources and access to healthcare workers in various countries, particularly in relation to mental health. There is an unequal distribution of professionally trained health workers globally, with countries facing higher disease burdens having significantly fewer resources. For instance, the African continent, experiencing 24% of the world's disease burden, struggles with access to merely 3% of trained health workers and < 1% of global financial resources (12). While the allocation of resources primarily favors infectious diseases, minimal attention is given to mental health, including cognitive disorders like dementia. Furthermore, considering mental health, other factors may interfere the diagnosis and management such as stigma and awareness (13), the limited infrastructure (associated with lack of human resources as already mentioned) (14), and the low funding and policy priority (15). African civilizations stigmatize mental health disorders, resulting in low awareness, therefore, mental health services are underutilized because stigma hinders people from seeking help (16). The lack of comprehensive policies and frameworks hinders mental health care development resulting in very limited hospitals and community centers tackling mental health issues. Furthermore, these services are often concentrated in cities, making rural access problematic. To address this health inequality, technological advancements, particularly mobile phone penetration, present an opportunity. Even in LMICs, mobile phone usage is prevalent, which can facilitate access to diagnostic and treatment applications for various physical and mental health concerns, including dementia (17).

Therefore, the objective of this paper is to emphasize the urgent need for the development of automated cognitive assessment methods that are culture-fair and demand only limited education in LMICs. By exploring alternative approaches that overcome the limitations of existing assessment tools, we aim to promote equitable access to accurate cognitive assessment, leading to more effective interventions and improved cognitive health outcomes for individuals in LMIC settings.

In the following sections, we will discuss the limitations of current assessment tools in more detail, examine the potential benefits of culture-fair and demanding only limited education automated cognitive assessment, and propose a framework for developing such tools in LMICs. Ultimately, our goal is to empower healthcare practitioners, researchers, and policymakers in LMICs to address the pressing cognitive health needs of their populations effectively.

## 2 Challenges in implementing traditional cognitive assessment in LMICs

LMICs face several challenges when implementing traditional cognitive assessment tools, impairing their ability to accurately evaluate cognitive functioning and identify impairments. These challenges include education disparities, cultural bias, language and communication barriers, and disparities in healthcare and resource availability.

### 2.1 Educational disparities

In many LMICs, there are significant educational disparities, with limited access to quality education, particularly in rural or marginalized communities (18). Traditional cognitive assessment tools often rely on literacy, numeracy, and educational background, making them inadequate for individuals with limited education. This creates a barrier to accurate assessment and leads to underdiagnosis or misdiagnosis of cognitive impairments (19).

### 2.2 Cultural bias

Cultural factors strongly influence cognitive processes and behavior. Traditional cognitive assessment tools developed in Western settings may not adequately account for the cultural diversity present in LMICs (20). Culturally biased test items and assessment procedures can lead to unfair evaluations, failing to accurately reflect individuals' cognitive abilities and impairments. For example, western examinations use English and use idioms and references recognizable to native English speakers. The Mini-Mental State Examination (MMSE) may use idioms like “a rolling stone gathers no moss,” which may confuse non-Westerners. Reading comprehension and numerical literacy are needed for tests like the Montreal Cognitive Assessment (MoCA) and MMSE. In areas with low literacy rates or educational backgrounds that differ from Western norms, test-takers may fail with these activities due to a lack of formal education. Some examinations use visual stimuli that may not be culturally meaningful across varied groups. Pattern recognition tasks containing Western symbols like clocks or animals may be alien to those from other cultures, resulting in inferior performance. Other limitations included the use of culturally specific terms or lists for memory tests. Words associated with Western food or daily objects may not resonate with people from different cultures, causing misunderstandings or poor memory performance without cognitive deficiencies.

This may undermine the validity and reliability of cognitive assessments in diverse populations.

### 2.3 Language and communication barriers

Many traditional cognitive assessment tools are administered in specific languages that might not be the primary language spoken by individuals in LMICs (21). Language barriers limit effective communication during assessments, resulting in compromised test performance and inaccurate cognitive evaluations (22).

### 2.4 Disparities in healthcare and resource availability

Healthcare disparities in LMICs, including limited funding, inadequate infrastructure, and a shortage of trained healthcare professionals, significantly impact the availability and accessibility of reliable cognitive assessment tools. The lack of resources hinders the implementation of comprehensive cognitive assessments, leaving many individuals without access to proper evaluation and subsequent interventions.

Furthermore, the availability of standardized assessment tools is often limited in LMICs due to cost, licensing, and language barriers. Many traditional cognitive assessments are developed and marketed primarily for high-income countries, making them less accessible and affordable for LMICs. This further widens the gap in cognitive healthcare services, depriving vulnerable populations of essential cognitive assessments.

The combination of educational disparities, cultural bias, and limited healthcare resources in LMICs creates a complex environment that poses substantial challenges to the implementation of traditional cognitive assessment tools. Addressing these challenges is critical to ensure equitable access to reliable cognitive assessments and improve cognitive health outcomes in LMIC populations.

## 3 Existing automated cognitive assessment tools

Several promising tools are already available, or currently under validation for assessment of cognitive functions in LMICs.

### 3.1 Technology-based assessments

Digital platforms, such as smartphone applications and computer-based tests, offer the advantage of flexibility and scalability. These tools utilize interactive tasks, games, and simulations to assess cognitive abilities (23, 24), minimizing the dependence on educational and cultural background. Mobile technologies offer significant advantages in the realm of cognitive impairment assessment, enabling repeated and continuous evaluations while discreetly collecting additional behavioral markers. This enhanced approach allows users to observe patterns over time and promptly detect acute changes that have historically posed challenges for identification. The potential opportunities in this domain encompass the utilization of cutting-edge mobile sensors and wearable devices, ensuring enhanced reliability and validity of mobile assessments. Moreover, there is a need to establish appropriate clinical applications for mobile assessment data and integrate person-centered assessment principles alongside digital phenotyping methodologies. Embracing these advancements holds the potential to revolutionize cognitive impairment assessment and support more personalized and effective healthcare approaches (25).

### 3.2 Non-verbal assessments

Non-verbal assessments, such as visual-spatial tasks, pattern recognition, and cognitive games, provide a means of evaluating cognitive functioning without relying heavily on language or literacy skills (26). These assessments can be particularly useful in populations with limited education or diverse linguistic backgrounds.

### 3.3 Performance-based measures

Performance-based measures focus on evaluating an individual's actual abilities and skills rather than relying on self-reported or subjective measures. These measures include objective performance tasks that assess cognitive domains such as attention, memory, executive function, and processing speed (27).

### 3.4 Limitations and applicability of tools in LMICs

While these culture-fair and limited education demanding assessment tools show promise, their applicability in LMICs is not without limitations and need consideration of several factors.

In the context of administering assessment tools, particularly in LMICs, it is crucial to consider the role of evaluators. The person responsible for evaluating individuals using these tools is often a trained health care worker from the community, rather than a professional healthcare worker specialized in cognition.

The widespread implementation of technology-based assessments relies on the availability of reliable internet connectivity and access to appropriate devices. In resource-constrained settings, particularly in rural areas of LMICs, limited technological infrastructure may hinder the widespread adoption of these tools (28–30).

Adapting assessment tools to different cultural contexts is essential to ensure their validity and reliability. The tools should be sensitive to cultural nuances, norms, and values has these parameters significantly influence cognitive assessment Some of the most important parameters included language and interpretation, conceptual understanding, time perception, and social desirability bias (31). For example, even with literal translations, language interpretation can vary. Verbal-based examinations may misread Western words, phrases, and idioms because they may not have equivalents in other languages (20). Secondly, colors, shapes, and items can have different meanings and various symbolic meanings throughout cultures, which may affect cognitive evaluation responses to visual stimuli. Another important aspect is that some indigenous and African civilizations view time cyclically or focus on the present, while Western societies view time linearly and forward. Planning, time estimation, and sequencing assessments can be affected by time perception. Finally, many Asian cultures respect authority, which might show in subtle or indirect communication. This may cause people to give answers they think the assessor wants, skewing cognitive testing results (32).

Efforts should be made to validate and adapt these tools across diverse populations, establishing their applicability in different cultural settings. This includes the development of normative data specific to various LMIC populations.

While non-verbal assessments reduce the reliance on language, literacy skills may still play a role in some tools. Adapting assessments to different languages and ensuring they are accessible to individuals with varying literacy levels is crucial for accurate cognitive evaluations.

### 3.5 Gaps in the current landscape of automated cognitive assessment for LMICs

The current landscape of automated cognitive assessment tools for LMICs reveals several gaps.

First, many of the tools reviewed lack extensive validation in LMIC settings. Hence, robust validation studies are needed to establish the reliability, validity, and cross-cultural applicability of these new informatics tools in diverse populations within LMICs. For example, even if digital health solutions have shown promising results in improving health outcomes through enhanced patient engagement and adherence to prescribed therapies (33), several factors limiting the implementation of such solutions have been identified. Challenges include technological infrastructure, cultural acceptance, and the need for training among healthcare providers. Second, normative data specific to LMICs are lacking and need to be developed. This is crucial for accurate interpretation of assessment results. Population-specific norms accounting for cultural, educational, and socioeconomic factors need to be established to ensure appropriate comparisons and interpretation of cognitive assessment outcomes. Finally, the scalability and affordability of these tools in LMICs need to be considered. Ensuring that the tools are cost-effective, easily accessible, and compatible with the available resources is vital for their implementation and sustainability in LMIC settings.

## 4 Strategies for developing automated cognitive assessment tools

Developing assessment tools that are culture-fair and demand only limited education requires careful consideration and collaboration among researchers, practitioners, and stakeholders (34, 35). Table 1 presents potential strategies for achieving this goal. By employing these strategies, we can develop culture-fair and limited education-demanding assessment tools that accurately evaluate cognitive functioning across diverse populations in LMICs. These tools will promote equitable access to reliable cognitive assessments, leading to improved healthcare outcomes, early detection of cognitive impairments, and targeted interventions tailored to the needs of individuals in LMIC settings (36).

**Table 1:** Various strategies for (1) developing automated cognitive assessment tools in LMICs and (2) address ethical and implementation challenges.

**Development of cognitive assessment tools**	
**Points**	**Description**
1. Multidisciplinary approach	Foster collaborations between experts in cognitive assessment, cultural anthropology, linguistics, and education to ensure a comprehensive understanding of cultural and educational factors that influence cognitive functioning. This multidisciplinary approach will help identify and address potential biases and develop assessment tools that are inclusive and sensitive to diverse populations.
2. Cultural adaptation	Modify existing assessment tools or develop new ones through a process of cultural adaptation and localization. This involves considering cultural norms, values, and practices during the design and implementation of assessments. This may include incorporating culturally relevant stimuli, adapting instructions and response formats, and considering the impact of cultural factors on cognitive processes.
3. Non-verbal performance-based measures	Emphasize the use of non-verbal and performance-based measures that rely less on language and educational background. This can include visual-spatial tasks, pattern recognition tasks, and performance-based activities that tap into cognitive domains without heavy reliance on verbal abilities or specific educational skills.
4. Technology-based assessment	Leverage the advancements in digital technology and mobile platforms to develop accessible and scalable assessment tools. Smartphone applications and computer-based tests can provide interactive and engaging tasks that are less influenced by cultural or educational factors. These tools can be designed to be language-independent, utilizing visual cues and intuitive interfaces.
5. Collaborative cross-cultural validation	Conduct rigorous validation studies across diverse populations within LMICs to establish the reliability and validity of culture- and education-independent assessment tools. This requires collaborative efforts involving researchers from different regions, cultural backgrounds, and language experts to ensure the tools are applicable and accurate across diverse populations.
6. Establishing normative data	Develop population-specific normative data that account for cultural, linguistic, and educational factors. This enables appropriate comparisons and interpretations of assessment results within specific LMIC populations. Collecting normative data across different regions and communities within LMICs is crucial to ensure accuracy and reliability.
7. Capacity building and training	Invest in training programs for healthcare professionals, researchers, and educators in LMICs to promote the understanding and implementation of culture- and education-independent assessment tools. This includes providing resources, workshops, and professional development opportunities to enhance skills in administering and interpreting these tools accurately.
8. User-friendly and accessible design	Ensure that the assessment tools are user-friendly, accessible, and suitable for individuals with varying levels of technological literacy and resources. Consider the availability of internet connectivity, device compatibility, and ease of administration to maximize the reach and adoption of the tools in diverse settings within LMICs.
**Addressing ethical consideration**	
**Points**	**Description**
1. Accessible and inclusive design	It is important to consider the unique needs, cultural backgrounds, and technological limitations of LMIC populations when designing cognitive assessment tools. It requires prioritizing user-centered design principles to ensure accessibility, usability, and inclusivity for diverse individuals, including those with limited technological literacy.
2. Collaboration and stakeholder engagement	It is essential to involve local communities, healthcare professionals, policymakers, and researchers in the development and implementation of cognitive assessment tools. Collaborative efforts are needed to ensure that assessments are culturally appropriate, address local concerns, and align with the specific needs of LMIC populations.
3. Data privacy and informed consent	It is also important to implement robust data privacy protocols, adhering to international standards and local regulations. Obtaining informed consent from individuals participating in cognitive assessments, clearly explaining the purpose, potential risks, and benefits of the assessments are needed. Providing individuals with control over their data and ensuring secure storage and anonymization practices are also essential.
4. Algorithmic transparency and accountability	Promoting transparency in the design and development of algorithms used in cognitive assessment tools is important. Thorough evaluation of algorithms for potential biases, regular auditing and monitoring of their performance, and striving for continuous improvement are also needed. Furthermore, it is also important to conduct external audits or independent evaluations to ensure fairness, reliability, and accuracy of the algorithms.
5. Local Capacity Building and Training	There is a need to invest in training healthcare professionals and researchers in LMICs on the ethical use and implementation of cognitive assessment tools. This includes educating them about potential biases, data privacy concerns, and the responsible deployment of these tools. Empowering local capacity ensures the ethical use of assessments and enhances the understanding of the implications and limitations of cognitive assessment technologies.
6. Regulatory frameworks and governance	Establishing clear regulatory frameworks and ethical guidelines are needed to govern the implementation and use of cognitive assessment tools in LMICs. Encouraging national or regional bodies is needed to provide guidance on ethical considerations, data protection, and quality assurance in cognitive assessment practices.

## 5 Ethical considerations

The ethical considerations associated with creating and using cognitive assessment tools that are fair across different cultures and need minimal instruction are of utmost importance in LMICs due to several reasons if we consider that health is a human right, therefore according to Ooms et al. “all people, regardless of where they live, should be entitled to the same collective efforts that can protect or improve their health” (37). The current dependence on conventional assessment techniques, which are frequently impacted by cultural and educational variables, maintains inequalities and undermines the precision of cognitive evaluations, giving rise to ethical problems regarding impartiality and fairness. The ethical obligation to ensure fair and consistent access to dependable cognitive evaluations is highlighted as a fundamental human entitlement, in line with the principle that every person, regardless of their location, should have access to collaborative endeavors that safeguard or enhance their wellbeing. Utilizing technology-based examinations provides a chance to tackle these ethical concerns, fostering equity by mitigating cultural bias and reliance on educational factors. Nevertheless, the use of these tools presents actual ethical dilemmas, encompassing concerns of infrastructure, connection, data privacy, and potential biases in algorithmic design. When implementing these tools in LMICs, it is crucial to prioritize ethical measures such as providing fair access, obtaining informed consent, being culturally sensitive, and protecting data privacy. The ethical aspects of this undertaking emphasize the requirement for a deliberate and cooperative strategy, giving importance to impartiality, inclusiveness, and regard for the varied populations being evaluated.

### 5.1 Equity in healthcare

Culture-fair and limited education-demanding assessments promote equitable access to reliable cognitive evaluations across diverse populations. By reducing the influence of cultural and educational factors, these tools aim to minimize disparities and ensure that individuals from different backgrounds receive fair and accurate cognitive assessments (38).

### 5.2 Early detection and intervention

Timely identification of cognitive impairments is crucial for implementing effective targeted preventive measurements or interventions (39, 40). Digital assessments can enable early detection of cognitive changes, allowing for targeted interventions to mitigate the impact of cognitive impairments on individuals' lives, enhance quality of life and decrease family and healthcare costs, making it ethically important, as highlighted by the WHO (41). Furthermore, cognitive tests should be accessible and fair regardless of cultural background or academic level to uphold healthcare equity. All people have the same chance of early diagnosis and treatment.

### 5.3 Monitoring cognitive health

Ethically, care should be constant and continuous, including cognitive health monitoring (42, 43). Regular assessment of cognitive functioning can aid in monitoring the progression of cognitive disorders, tracking treatment effectiveness, and adjusting interventions accordingly. Regular assessments provide a standardized and objective means of monitoring cognitive health across diverse populations in LMICs (44, 45).

### 5.4 Potential biases in algorithmic design

Algorithmic bias can emerge if cognitive assessment tools are developed using datasets that are not representative of the diverse populations in LMICs. Biased algorithms may perpetuate inequalities, lead to misdiagnoses, or result in differential access to resources and interventions (46). Additionally, cultural biases in assessment tasks or scoring mechanisms can unfairly disadvantage certain groups.

## 6 Implementation challenges

The implementation of automated cognitive assessment tools in LMICs raises important practical considerations. Addressing these challenges is crucial to ensure the ethical and responsible deployment of cognitive assessment tools in LMICs.

### 6.1 Infrastructure and connectivity challenges

LMICs often face limitations in technological infrastructure and internet connectivity, particularly in rural or underserved areas. Implementing technology-based assessment tools requires reliable access to devices, internet connectivity, and a power supply (47). Unequal access to technology can exacerbate existing health disparities and impede the equitable distribution of cognitive assessments (48).

### 6.2 Data privacy and security

The collection, storage, and management of sensitive personal data during cognitive assessments raises concerns about data privacy and security. Safeguarding individuals' confidentiality and protecting their personal information are of paramount importance (49). Additionally, informed consent procedures should be robust, ensuring that individuals understand the purpose, risks, and benefits of cognitive assessments and have control over their data.

## 7 Measures to address ethical and implementation challenges

When developing novel solutions for cognitive assessment in LMICs, several crucial points must be taken into consideration. These points include accessible and inclusive design, collaboration and stakeholder engagement, data privacy and informed consent, algorithmic transparency and accountability, local capacity building and training, and the establishment of regulatory frameworks and governance, all of which are presented in Table 1. These considerations are essential to overcome challenges related to cultural, technological, and ethical factors in LMICs while ensuring the reliability and fairness of cognitive assessment tools.

## 8 Conclusion

Developing culture-fair and limited education demanding assessment tools for LMICs is vital for equitable access to reliable cognitive evaluations. Although promising tools have been identified, further research, validation, and adaptation are needed to address limitations and enable widespread implementation in LMIC settings.

These tools have the potential to significantly impact public health, enabling early detection and intervention for cognitive impairments. Timely identification allows for appropriate care, slowing cognitive decline, and easing the burden on healthcare systems.

Culture-fair and limited education demanding assessments also enhance accurate diagnosis and monitoring of cognitive disorders, tailoring interventions to individual needs in LMICs. This personalized approach leads to improved cognitive health outcomes.

To fully harness their potential, rigorous validation and collaboration between stakeholders are necessary. Adapting to diverse cultural contexts and developing population-specific norms ensures reliability and validity. Overcoming resource constraints and implementation challenges will ensure scalability and sustainability.

By embracing these tools, we advance public health, reduce disparities, and empower individuals to lead fulfilling lives in LMICs. Bridging the gap in cognitive assessment accessibility requires concerted efforts and a commitment to equity in healthcare.

## Author contributions

DG: Writing – review & editing, Writing – original draft, Conceptualization. DL: Writing – review & editing, Supervision. TA: Writing – review & editing, Supervision. BB: Writing – review & editing, Writing – original draft, Supervision, Funding acquisition, Conceptualization.
